# Improvement of personality functioning in patients with personality disorders: a comparative study of mentalization-based treatment versus non-manualized treatments

**DOI:** 10.3389/fpsyt.2026.1831782

**Published:** 2026-06-22

**Authors:** Kjetil Bremer, Geir Pedersen, Andreas Ekberg, Katharina T. E. Morken, Helene A. Nissen-Lie, Eileen Oftedal, Theresa Wilberg, Elfrida. H. Kvarstein

**Affiliations:** 1 Section for Treatment Research, Department of research and Innovation, Division Mental Health and Addiction, Oslo University Hospital, Oslo, Norway; 2 Department of Psychology, Faculty of Social Sciences, University of Oslo, Oslo, Norway; 3 Quality Laboratory of Psychotherapy, National Advisory Center for Substance Use and Addictive Disorders, Severe Concurrent Mental Health Disorders and Personality Disorders, Oslo University hospital, Oslo, Norway; 4 Nydalen District Psychiatric center, Division Mental Health and Addiction, Oslo University Hospital, Oslo, Norway; 5 Addiction and Substance Use Clinic, Haukeland University Hospital, Bergen, Norway; 6 Department of Biological and Clinical Psychology, University of Bergen, Bergen, Norway; 7 Group Outpatient Clinic, Stavanger District Psychiatric Center, Division of Adult Mental Health Care, Stavanger University Hospital, Stavanger, Norway; 8 Department of Caring and Ethics, Faculty of Health Sciences. University of Stavanger, Stavanger, Norway; 9 Institute for Clinical Medicine, Faculty of Medicine, University of Oslo, Oslo, Norway

**Keywords:** mentalization based therapy, non-manualized psychotherapy, personality disorder, personality functioning, psychotherapy, avoidant personality disorder, borderline personality disorder, group therapy

## Abstract

**Objective:**

Among evidence-based treatments for personality disorder (PD), mentalization-based treatment (MBT) represents a manualized, long-term treatment. Both MBT and non-manualized psychotherapeutic treatments (NMT) are currently applied for patients with PDs in Norway. This clinical population is characterized by varying impairment of personality functioning (PF). Few have investigated how MBT and NMT are suited for the heterogeneity of personality problems presenting to health services and how they compare. This study aimed to investigate 1) characteristics of patients admitted to MBT versus NMT, 2) longitudinal change in PF among patients in MBT versus NMT, and 3) associations between initial PD status and outcome differences in MBT and NMT.

**Methods:**

The current study is observational, reflecting regular outpatient PD health services. Data was retrieved from a Norwegian quality registry (Network for Personality Disorders, period 2017-2024) and included all patients with information on baseline PD assessment (SCID-5-PD), treatment specification (i.e., MBT or NMT), and self-report data on PF outcome (global level of personality functioning: LPFS-BF, and specific personality problems: SIPP-SF, N = 954, N_MBT_= 565, N_NMT_= 389). Longitudinal estimations were based on linear mixed models.

**Results:**

Patients admitted to MBT treatment had more severe personality pathology, more often a Borderline PD (BPD) diagnosis, and a higher symptom burden compared to NMT. Treatment condition explained little longitudinal variation and improvement of all PF aspects during treatment was comparable. Effect sizes ranged from medium to large in both treatment groups. Increasing number of borderline and narcissistic PD criteria were independently associated with greater improvement in MBT versus NMT. Poorer improvement in MBT was associated with a higher number of avoidant PD (AvPD) criteria.

**Conclusion:**

Admittance to MBT versus NMT appeared largely matched to severity and type of PD. Clinically relevant improvements were found in both MBT and NMT. Improvement rates were larger in MBT for patients with BPD, more severe PD conditions, and comorbid narcissistic traits. Further development of effective treatments for AvPD is needed.

## Introduction

Personality disorders (PDs) are common mental health disorders, as reflected in the community (1) and in clinical samples (2). Typically, PDs can represent a substantial burden both to the individual, their social life, and to society (3–5). Psychotherapy is the first line treatment (6) and there are several manualized treatments showing evidence of its effectiveness (7–9). The present study investigates patients admitted to personality focused treatments conducted within routine mental health services, offering both manualized and non-manualized treatments.

In line with the transition from categorical to dimensional understanding of personality difficulties, the diagnostic manuals of both ICD-11 (10) and DSM-5 AMPD (11) describe PD as a combination of impaired personality functioning (PF) and specification of maladaptive personality traits. PF is in DSM-5 AMPD characterized by dimensions of the self (identity and self- direction) as well as interpersonal relationships (empathy and intimacy). These aspects are understood along a continuum that spans from optimal functioning to severe pathology (11). High levels of PF are associated with a stable sense of self, ability to make meaning of oneself and others, as well as to establish and pursue goals, and develop and maintain nurturing relationships. PF is found to be relevant for developing flexible coping strategies and well-being (12) as well as for a healthy psychosocial functioning (13, 14). Increased risk of substance use, harm to self or others, dissociative symptoms and suicidality are associated with more impaired PF (15). Based on recent meta-analytic studies, it is recommended that future treatment research on PD includes a dimensional approach (16). PF is furthermore advanced as a nonspecific marker of health vulnerability, risk, and service needs (15). In the current study, PF is the main focus of investigation.

Mentalization - based treatment (MBT) is one of the recommended manualized therapy methods for borderline PD (BPD) and has been implemented in several countries, including Norway (17). Mentalization is a multifaceted construct briefly described as the capacity to reflect and make sense of oneself and others in terms of their intentions, feelings and behaviors (18). Mentalizing involves the flexible ability to modulate and integrate mental states across different dimensions, including feelings and cognitions, oneself and others, registering both internal and external attributes, and the ability to detect implicit assumptions and make them explicit (ibid). Increased ability to mentalize is argued to be a desired outcome across therapy models and different disorders (19). MBT essentially, aims to increase patients’ mentalizing capacities. The treatment is based on a structured framework combining manualized psychoeducation, individual and group therapy formats (20–23). The manualized approach specifies case formulations, crises management, teamwork, and regular supervision for therapists.

Despite the heterogeneity of PDs in clinical populations, the majority of PD treatment research has historically been on BPD (6). Knowledge of treatment for other PDs is sparse but has received increased interest in recent years (24). MBT was originally invented for patients with BPD. Adaptations of MBT to other types of PDs have been developed for both narcissistic, antisocial and avoidant PDs (25), but have not been broadly tested. In the present study sample, MBT as originally designed for BPD, was the manualized PD treatment approach.

Substantial variation exists in how psychotherapy is organized and delivered across institutions and locations. In a recent study based on Norwegian PD health services, nearly half of the patients received one of the manualized PD treatments (26). For the remaining sample, treatment was not standardized with respect to manuals, intensity, and duration. Psychodynamic group therapy was the most prevalent, and combinations of treatment included individual therapy, and combinations with other groups, including cognitive approaches or focused on physical exercise or bodily awareness. Former studies of non-manualized psychodynamic group-based therapies for patients with PD before the advent of manualized PD therapies, have reported clinically relevant improvements of symptom burden, and five-year effect sizes were found to be in the large range, with approximately half of the patients achieving non - clinical levels of functioning (27, 28). However, there were notable drop-out rates for BPD patients and generally poorer outcomes among patients with avoidant PD (AvPD). In the present study the frequently applied non - manualized treatment (NMT) are defined as local variations of different “bona fide” PD focused treatment approaches not structured by guidelines and manuals.

In clinical samples, PD conditions vary, ranging from milder personality problems to complex cases with comorbidity of different PDs and other mental health disorders. Challenging the overrepresentation of BPD research, a study of treatment seeking patients with PD, demonstrated that only 31% qualified for BPD, whereas AvPD as the most frequent disorder (39%), and 24% had more complex PD comorbidity (26). Former studies of PD treatments have recommended manualized therapy programs for severe PD conditions and simpler stand-alone group psychotherapy formats for patients with milder personality problems (29, 30). Tailored, personalized organization of treatment is increasingly focused and clinical management matched to the patients level of PF has been recommended (15). Studies of MBT have demonstrated that this manualized approach was particularly beneficial for BPD patients with clinically severe conditions (31–33). MBT as well as other evidence-based treatments specifically designed for BPD (including Dialectical Behavior Therapy (DBT; (34)), Schema-Focused Therapy (SFT; (35)), and Transference-Focused Psychotherapy (TFP; (36)) have generally shown positive results for patients in this target group (37). These treatments have different theoretical traditions and types of interventions, but still a noteworthy overlap in terms of content and organization where they all have a primary PD focus and varying combinations of psychoeducation, individual and group therapy formats (e.g. (38, 39)). It is still an open question how PF interacts with personality traits, relational patterns, and treatment processes (40). Guided by the enduring question “what works for whom” (41), the present study explores the patients’ level of PF and how it is managed within heterogeneous clinical samples of mental health services.

The present study is part of a large, longitudinal, multicenter research project (TREATPD) based on clinical data from the quality register of the Norwegian Network for PD (The Network) – a cross-regional quality and research collaboration of PD treatment units within specialist mental health services (42). The overall aim of TREATPD is to evaluate the utility of PD treatment provided within specialist mental health services. TREATPD explores treatment seeking patients and their course through treatment and covers a range of perspectives (5, 26, 43, 44).

The overall aim of the present study is to investigate the development of PF for patients seeking PD treatments in light of the chosen type of treatment and the type and severity of personality problems apparent on admission to treatment. Our research questions were explorative and included I: How do patient characteristics differ in MBT versus NMT? II: How does change in PF during treatment differ in MBT versus NMT? III: How is PD severity and type associated with longitudinal course of PF in MBT versus NMT?

## Methods

### Design

The present study is explorative and has an observational, naturalistic longitudinal design. Data was extracted from the quality register of the Network.

### Treatment settings

Treatment units within the Network are all specialized group-based outpatient clinics for adults, part of the public specialist mental health and addiction services in Norway. Their primary target group is people with PDs and personality-related difficulties (42). The treatment units represented a second line within the mental health and addiction service, patients were referred after a brief, first line, general mental health assessment. In the given study period, Network collaborations were implemented across health regions in Norway, but such PD treatment units or equivalent specialized PD treatments were not available within all mental health care centers in Norway.

### Sample

The extracted data included all patients admitted to and discharged from treatment within the Network during the period 2017–2024 who had information on baseline PD assessment (SCID-5-PD), treatment specification (MBT or NMT) and longitudinal self-reports of PF (N_LPFS-BF_= 942, N_SIPP-SF_= 934). MBT was the most frequent manualized approach. Patients who had received other manualized treatments (DBT; N = 30, SFT; N = 114, Metacognitive Interpersonal Therapy (MIT; 45); N = 39) were not included in the study sample, as well as patients who had received MBT psychoeducation as a stand-alone treatment (N = 155). Sample selection included patients from 16 treatment units. See **Table 1** below for information on the sample.

**Table 1:** Baseline status of demographics, personality functioning and symptom distress.

Demographics, PF and symptom distress	MBT		NMT		Differences	
	Frequency %	Mean (SD)	Frequency %	Mean (SD)	p	η^2^
Age		29 (8.1)		30 (8.1)	0.005	0.009
Female	79		75			
Former outpatient treatment	81		81			
Former inpatient admissions	38*		26			
Age < 18, first treatment	69*		61			
Borderline PD	55*		16			
Avoidant PD	38		45			
Schizoid PD	1		1			
Schizotyp PD	0.2		0.3			
Paranoid PD	10*		5			
Antisocial PD	3		1			
Narcissistic PD	2*		0,3			
Histrionic PD	1		0			
Dependent PD	9*		4			
Obsessive compulsive PD	6		7			
NOS PD	11*		17			
Not PD diagnosis	6*		19			
Total SCID - 5 PD crit.		13.2 (6.2)		10.2 (6.0)	<0.001	0.09
Number of antisocial adult criteria		0.4 (0.9)		0.1(0.5)	<0.001	0.02
Number of narcissistic criteria		0.4 (1.1)		0.1 (0.5)	<0.001	0.02
Number of borderline criteria		4.3 (2.7)		2.1 (2.2)	<0.001	0.16
Number of avoidant criteria		2.8 (2.2)		3.1 (2.2)	0.03	0.005
Number of paranoid criteria		1.4 (1.6)		0.9 (1.2)	<0.001	0.03
Number of dependent criteria		1.6 (1.7)		1.2 (1.4)	<0.001	0.02
LPFS-BF		20.7 (5.9)		18.1 (6.4)	<0.001	0.01
Self-harm ever	76*		63			
Self-harm last 6 months	46*		28			
Suicide attempt ever	49*		36			
Suicide attempts last 6 months	16*		8			
Mood disorders	68		75			
PTSD/Dissociative disorder	16		8			
OCD	6		4			
Other anxiety disorders	41*		30			
Substance use disorders	15*		4			
Eating disorders	12		7			
ADHD	9		7			
Somatization disorder	3		3			
Psychosis/Autism disorders	1		1			
Number of symptom disorders		1.4 (1.6)		1.0 (1.2)	*ns*	0.002
PHQ-9		19.1 (5.0)		17.6 (5.0)	<0.001	0.02
GAD-7		14.0 (4.4)		12.7 (4.5)	<0.001	0.02

Multivariate analysis of variance was used to analyze differences between the two treatment conditions on multiple continuous variables. Effect sizes are given by Partial Eta Squared (η^2^), where η^2^ = 0.01 indicates a small effect. η^2^ > 0.06 medium to large effects. * indicates p<0.01, chi square tests for comparison of categorical data. Non-significant differences are indicated by ns (p>0.5).

### Procedures

Data collection is based on administrative routines of the Network comprising a set package of patient- and therapist reports administered locally at baseline (before starting treatment) and for outcome measures, every 6 months of treatment. Patient-reports included psychosocial status, measures of PF and symptom distress. Allocation of patients to MBT versus NMT was primarily based on clinical judgment within each treatment unit. Availability of MBT programs was high with only one of the 16 included treatment sites without MBT and 12 of the 16 sites offered both MBT and NMT. Therapists reported information from diagnostic interviews at baseline, and type of treatment applied every six months of treatment. At the end of treatment therapists also reported on treatment completion. “Drop-out” was then defined as terminating treatment before planned, against the therapists advice. The study follows principles of intention to treat. All patients admitted to treatment were included in data analyses irrespective of how they completed treatment.

### Measures

#### Assessment of diagnoses

Diagnostic interviews were performed by trained clinicians in the Network on referral to treatment before intake was decided (baseline) Semi-structured assessment interviews included the Mini International Neuropsychiatric Interview (46) for symptom disorders and the Structured Clinical Interview for Personality Disorders (SCID-5-PD) (47). At regular intervals during the study period therapists in the Network received training in clinical evaluation and diagnostic competence of personality pathology in workshops given by an experienced psychiatrist (last author) to ensure diagnostic reliability and calibration. During COVID-19, workshops were held digitally. All diagnoses given were confirmed by a specialist in clinical psychology or psychiatry at each unit.

#### Assessment of psychosocial status

Patient self-reports on psychosocial status comprised items designed for the Network covering age, gender, former treatment experience, former self-harm and suicide attempts, and were administered at the baseline assessment (see **Table 1**; **Supplementary File 1**).

#### Repeated assessment of personality functioning, baseline and every 6 months of treatment

##### Levels of personality functioning scale brief form

LPFS-BF-2.0 is a 12-item questionnaire developed to address intrapsychic and interpersonal personality problems in the Alternative Model for PD (AMPD) in DSM – 5 Section III (48). It has shown sound internal consistency applicable across diverse populations (49), cultures (50) and age (51) with good convergent and discriminant validity (52). Studies indicate acceptable construct validity, sensitivity to change (48) and ability to distinguish between those above and below clinical cutoff (52) and moderate to good support for the use of total scores (53). Patients were asked to fill in the extent to which the questions applied to their current condition (“no fit”, “fit a little”, “fit often”, “fit completely”). Items were scored on a 4- point scale from 0-3, sum scores range from 0 – 36, LPFS -BF > 14 representing clinically relevant personality dysfunction likely corresponding to personality disorder (54).

##### Severity indices of personality problems - short form

SIPP-SF is a self - report measure using 60 of the 118 items from SIPP 118 (55). SIPP-SF is found to be a valid and reliable measure of PF of DSM- 5 AMPDs self -domain (identity and self-direction) and interpersonal domain (empathy and intimacy) (56). SIPP - SF has shown the same factor - structure as the full version, with five domains (57) and applicable across different ages (56, 58). The SIPP-SF domains are *Self-control* (“the capacity to tolerate, use and control emotions and impulses”), *Identity integration* (“coherence of identity; the capacity to see oneself and one’s own life as stable, integrated and purposive”), *Relational capacities* (“capacity to care about others as well as feeling cared for, to be able to communicate personal experiences, and to hear and engage with the experiences of others, though not necessarily in the context of long-term, intimate relationships”), *Social concordance* (“ability to value someone’s identity, withhold aggressive impulses towards others and to work together with others”) and *Responsibility* (“the ability to set realistic goals, and to achieve these goals in line with the expectations generated in others”). Patients were asked to fill in the extent to which they agreed to the questions, considering the last three months. Statements were rated on a 4-point Likert scale ranging from “fully disagree” (1) to “fully agree” (4). Higher T- scores on domains indicate more adaptive personality capacities. Scores are rated as “very low” (<30), “low” (30–39), “mediate” (40–59), “high” (60–69), and “very high” (>70) (59).

#### Assessment of self-harm and suicide attempts

Items were designed for the Network. We report patient self-reports recording status the last six months. These were administered at baseline and in the last phase of treatment (see **Supplementary File 1**).

#### Additional repeated assessment of mental health symptoms (baseline and every six months)

The Patient Health Questionnaire, Depression (PHQ-9) is a patient self-report on depression symptoms (9 items, 0–3 scale) (60). Sum scores ≥ 10 indicate clinically relevant depressive symptoms (61). The Generalized Anxiety Disorder-7 (GAD-7) is a patient self-report of anxiety symptoms with seven items (0–3 scale) (62). Sum scores ≥ 10 indicate possible anxiety disorder (63). GAD-7 and PHQ-9 were registered by patients at baseline and every 6 months of treatment.

### Therapists

The therapists worked in multidisciplinary teams, professions included mainly psychologists, psychiatric nurses, psychiatrists, social workers and resident doctors. Specific information on therapists is not included in the quality register, which precludes reporting the exact numbers of professions in the therapist sample. However, in a recent, independent survey of therapists in the Network in the same study period (response rate 56%), 72% were female, years of clinical experience was <5 (25%) and >20 (22%) (64). The most frequent psychotherapy training was in psychodynamic group psychotherapy (53%), MBT-group (34%), MBT-individual (22%) and “other” (39%) (Ibid.). As a quality and research collaboration, therapists within the Network were invited to annual conferences, regular workshops and seminars focusing on PD assessment and treatment arranged by the Network.

### Therapeutic approaches

The therapists registered what kind of treatment was delivered in the last 6 months. The options for registration were manualized treatments (MBT, DBT, SFT or MIT), or other (non-manualized psychotherapeutic treatments). In accordance with the aims of this study, we chose to categorize therapeutic approaches that were either manualized (including only MBT), or non-manualized (NMT). NMT is not informed by manuals, it is not a clearly defined therapy program for PD specific problems and does not include a standardized format. The different therapies in this category were typically more varied between sites with different individual and group therapies (**Table 2**).

**Table 2:** Specification of non-manualized treatments (NMT).

NMT n = 389	
Group therapy	85%
Psychodynamic	58%
Art therapy	4%
Stabilizing trauma course	2%
Cognitive social anxiety	2%
Physical exercise	3%
Body oriented	8%
“not specified”	8%
Individual therapy	56%
Psychodynamic	30%
Cognitive	4%
Supportive	6%
Affect- consciousness	2%
Exposure trauma	1%
“not specified”	13%

#### Mentalization-based treatment

Based on therapist registered forms, 59% of the patients in the study sample were categorized as receiving MBT. MBT was based on the available MBT manuals in Norwegian language. The format was long-term (1–3 years) average duration 24 months (SD: 12) and included four integrated components: a preparatory psychoeducational group (10–12 weekly sessions), case formulations, individual therapy, and group therapy (20–22). The individual and group therapy were conjoint, initially offered weekly, with decreasing intensity of individual therapy as involvement in group therapy increased (first year weekly, second year every other week). To further support MBT implementation and need for supervision and calibration of MBT principles across units, the Network arranged supervisory workshops every six months for MBT therapists. The workshops covered the whole study period.

#### MBT organizational quality

Information on organizational quality is based on telephone interviews with leaders in the treatment units in the Network (performed by the first author 2024 - 25). The interviews were informed by the MBT quality manual which states quality standards for MBT implementation (65). The quality manual does not give cut-off levels for above or below “good enough” MBT implementation standards. An overall evaluation of information from the sites in the present study indicated an implementation of MBT in the Network covering major quality standards (65): 1) All MBT teams were multidisciplinary, 2) team sizes included at least four therapists, seldom above ten, 3) caseloads were fewer than 18 individual consultations a week and most often 1–3 group sessions per week. 4) All MBT units used case formulations and crises plans, offered a combination of psychoeducation, individual and group therapy, applied at a regular level of at least 18 months. 5) Individual and group therapists formed “mini – teams” round each patient, with the individual therapist being the principal therapist, 6) All units had regular supervision (76% weekly), mainly based on video recorded therapy sessions (72%), and most often in a group format (82%). 7) Approximately 3/4 of the therapists had MBT training, and 2/3 of the teams had MBT qualified supervisors. Deviation from the quality standards included process supervision or intervision teams (only half of the units), and regular evaluation of the MBT supervision (none of the units). The overall standard of MBT implementation was on level with standards reported in a former MBT study at a highly specialized research clinic (66).

#### Therapist adherence to MBT

Assessment of therapists’ in-session adherence to MBT was based on video-taped sessions evaluated by trained scorers at the Quality Laboratory of Psychotherapy, within the National Advisory Unit for Personality Disorders, Oslo University Hospital. The laboratory is a regular supervisory service (funded by regional health authorities), designed for therapists treating patients with PD within mental health services. MBT adherence scores reported in this study are based on videos evaluated as regular clinical procedures using validated rating scales available in Norwegian language in the study period (67, 68). The data was collected retrospectively and included only information on scores, not specific therapists, patients or units. Data included a total of 42 tapes from 10 different sites in the Network scored within the study period (MBT-group therapy scores of 28 tapes from 9 sites (mean score: 4,7. SD 0,7) and MBT- individual therapy scores of 14 tapes from 2 sites (mean score: 4,5. SD: 1,2)). Score range from 1-7, and above 4 indicate that the interventions are sufficiently in accordance with the MBT approach.

### Non-manualized treatments

Based on therapist registered forms, 41% of the patients in the study sample were categorized as receiving NMT (See **Table 2**). The different local treatment approaches that were registered in this category included both individual and group-based treatments, average treatment duration, 21 months (SD: 12). In NMT the dominating approach was psychodynamic group psychotherapy (58%), either as a stand-alone treatment or in combination with other groups or individual therapy. The psychodynamic group therapies were “slow- open”, and had weekly sessions lasting for 1,5-hour, with a maximum of 8 patients. The most predominant individual therapy was psychodynamic (30%). In total, 83% of the patients in NMT who had individual consultations (of any approach), also attended group therapies. There were no measures of adherence to treatment models of NMT.

### Statistical analysis

Analysis was performed using SPSS Statistics for Windows, Version 30 (69).

#### Longitudinal models

The main statistical method was Linear mixed models (LMM) (70, 71). Self-reported assessment of PF (LPFS-BF sum-score and the five SIPP- SF domains) were analyzed as dependent variables. Treatment (MBT or NMT) was treated as an independent variable (predictor). Time (months from baseline) was modelled as a continuous variable. In accordance with log likelihood estimations, the best-fitted model included linear time, random intercept and slope, and an unstructured covariance matrix. Additional corresponding models included the dependent variables PHQ-9 and GAD7. For further model specification and interpretation, see **Supplementary File** (**Supplementary File 2**). The models are generally specified in the formula: PF = Fixed Intercept + (Fixed Effect: Treatment) + (Fixed Effect: Time) + (Random Intercept) + (Random Slope * Time) + Error. Differences according to PD status (type and severity of PD) were further investigated in separate PF models added as fixed effects: PD variable * Treatment + PD variable * Treatment * time. Due to considerable comorbidity of PD features, these models included 1) the two most frequent PD categories (BPD and AvPD), 2) the total number of PD criteria and 3) independent contributions of specific PD criteria based on preliminary, exploratory LPFS-BF models investigating independent effects of all the different PD criteria. PDs with non-significant independent effects were not included in further analyses.

#### LMM based effect sizes

For illustration of longitudinal change, we present effect sizes, Cohen’s *d* based on LMM predicted values in models including treatment condition and treatment duration. Small effect size: *d* = 0.2, medium *d* = 0.5, large *d* = 0.8 (72). We report 0–24-month effect sizes as the closest approximation to times of assessment and the mean treatment durations (overall mean treatment duration 23 months (SD 12).

#### Models controlling for possible bias

Control analyses included models investigating possible impacts of mood disorder, age and gender. The data-collection was limited to the treatment period and shorter treatment duration naturally caused fewer assessments. Since treatment duration varied within the sample, its association to outcomes was investigated as a separate predictor in the main analyses as a control variable/covariate. Main analyses were also replicated in subsamples including only patients with PD and subsamples excluding a small proportion who received both MBT and NMT in the study period (6%). Control analyses are elaborated in **Supplementary Material** (**Supplementary File 3**).

#### Longitudinal data quality

Mean number of repeated LPFS-BF/SIPP-SF self-reports per individual was 2.6, 95% had the baseline LPFS-BF assessment and 96% the SIPP-SF assessment before starting treatment. Among patients with treatment duration > 12 months (n = 761) the mean number of LPFS-BF assessments was 3.8 and SIPP SF assessments was 2.9. To further investigate the possible longitudinal bias due to missing data, a variable counting the number of assessment points was investigated as a longitudinal predictor in separate models (73). These results did not render significant differences in change over time (*p*_LMM <_0.05).

#### Other descriptive statistics

Multivariate analysis of variance (MANOVA) was used to analyze differences between the two treatment conditions on multiple continuous variables. Other comparisons of categorical data were made using crosstabs (Chi square) for estimation of effect sizes.

### Ethics

All data in the Network quality register is anonymous and cannot be traced back to the contributing units or patients. Contribution to the quality register is based on the patients’ written consent. Data collection and transferal procedures were approved by local Data Protection Officers at each of the units. The security procedures of the quality register are approved by local Data Protection Officers at the responsible research center (Oslo University Hospital). Since data in the quality register is anonymous, further approvals from Regional Committee for Medical Research and Ethics were not required.

## Results

### Patient characteristics

**Table 1** demonstrates demographics, psychosocial and diagnostic status on referral to treatment (baseline) for patients admitted to MBT and NMT. Overall, the majority met criteria for a PD, other comorbid mental health disorders were frequent, former use of mental health services was prevalent, often starting in adolescence. Comparing MBT and NMT, patients in MBT had significantly younger age of first contact, more frequent former inpatient admissions, higher number of SCID-5-PD criteria, more frequent self-harm and suicide attempts, and significantly higher proportions of comorbid anxiety and substance use disorders (**Table 1**). In NMT 19% and in MBT 6% of the patients did not qualify for a full PD diagnoses. Among specific PDs, AvPD and BPD were most frequent in both MBT and NMT, though BPD was more dominant in the MBT group. Among less frequent PDs, narcissistic, paranoid and dependent PD were more frequent in MBT. A notable proportion qualified for any narcissistic PD criteria, and this was higher in MBT group (MBT: 19%, NMT:10%, *p* = 0.004). Avoidant PD was slightly more frequent in NMT (*p* = 0.03). Levels of LPFS-BF and most SIPP-SF domains indicated greater impairment of PF in MBT for all aspects except SIPP-SF Relational capacities (p _LMM <_0.001, **Tables 3** and **4**, explained baseline variation: mean 5%, highest, SIPP-SF Self-control: 9% and lowest, SIPP-SF Relational capacities: 0%). Levels of GAD7 and PHQ-9 were higher in MBT (p_LMM_ <0.001, explained baseline variation: PHQ-9: 4%, GAD-7: 5%).

**Table 3:** Longitudinal trajectories of personality functioning.

Model N = 954	Predictor	Moderator	Fixed effects				Variance components		Model fit
			Intercept estimate (SE)	*p*	Slope estimate (SE)	*p*	Intercept variance	Slope variance	AIC
LPFS-BF			19.6 (0.19)	<.001	-0.062 (0.01)	<.001	26.6*	0.036*	15396
			Δ Est. (SE)	*p*	Δ Est. (SE)	*p*	Explained variance %		ΔAIC
LPFS-BF	ΔTreatment		-2.56 (0.4)	<.001		ns	6	0	41
LPFS-BF	ΔTreatment	PD crit	0.24 (0.02)	<.001		ns	14	0	103
LPFS-BF	ΔTreatment	AVPD	5.4 (0.6)	0.002		ns	15	0.035	102
LPFS-BF		BPD	4.0 (0.5)	<.001	-0.07 (0.03)	0.03			
LPFS-BF	ΔTreatment	BPD crit	0.6 (0.07)	<.001	-0.01 (0.004)	0.04	17	0.034	119
		NPD crit	0.6 (0.2)	0.01		ns			
		AVPD crit	0.3 (0.09)	0.005	0.01 (0.005)	0.03			

Demonstrates LMM estimations with standard errors (SE) for baseline levels (intercept estimate), and monthly change-rate (slope estimate), and corresponding variance estimates for the dependent variable LPFS-BF. Significant estimates of variation are indicated by *(p<0.01). Goodness of model fit is indicated by Akaike Information Criterion (AIC), where smaller is better. Predictor analyses include the difference (Δ estimate) between treatment (ΔTreatment = Non-Manualized Treatment versus Mentalization-based Treatment), total number of PD criteria (crit), and the specific personality disorders, avoidant (AvPD) and borderline (BPD) in separate models and models controlling for the two. Interaction between ΔTreatment and PDs (moderators) additionally includes a model with the number of criteria within significant specific PDs. Non-significant differences are indicated by ns (*p*>0.05).

**Table 4:** Longitudinal trajectories of specific personality problems.

Model N=934	Predictor	Moderator	Fixed effects				Variance components		Model fit
			Intercept estimate (SE)	*p*	Slope estimate (SE)	*p*	Intercept variance	Slope variance	AIC
Self-control			30.8 (0.5)	<.001	0.33 (0.02)	<.001	160.1*	0.131*	18459
Identity			20.7 (0.3)	<.001	0.39 (0.02)	<.001	77.1*	0.135*	18245
Social			39.9 (0.4)	<.001	0.18 (0.02)	<.001	153.8*	0.123*	18439
Relational			32.9 (0.3)	<.001	0.19 (0.02)	<.001	72.7*	0.063*	17334
Responsible			33.6 (0.2)	<.001	0.22 (0.02)	<.001	144.1*	0.040*	18331
			Δ Est. (SE)	*p*	Δ Est. (SE)	*p*	Explained variance %		ΔAIC
Self-control	ΔTreatment		-7.9 (0.9)	<.001		ns	9	0	76
Self-control	ΔTreatment	PD crit	-0.7 (0.05)	<.001		ns	18	0	158
Self-control	ΔTreatment	AVPD		ns	-0.17 (0.05)	<.001	30	9	269
		BPD	-13.8 (1.2)	<.001	0.20 (0.05)	<.001			
Self-control	ΔTreatment	BPD crit	-2.5 (0.1)	<.001	0.04 (0.01)	<.001	33	12	298
		NPD crit		ns		ns			
		AVPD crit	0.5 (0.2)	<.001	-0.04 (0.01)	<.001			
Identity int.	ΔTreatment		-3.2 (0.7)	<.001	-0.11 (0.05)	0.02	3	1	26
Identity int.	ΔTreatment	PD crit	-0.33 (0.04)	<.001		ns	8	0	62
Identity int.	ΔTreatment	AVPD	-4.9 (0.8)	<.001	-0.20 (0.1)	<.001	11	4	90
		BPD	-4.3 (0.7)	<.001		ns			
Identity int.	ΔTreatment	BPD crit	-0.6 (0.1)	<.001		ns	11	4	87
		NPD crit		ns		ns			
		AVPD crit	-0.90 (0.2)	<.001	-0.04 (0.01)	<.001			
Social conc.	ΔTreatment		-6.4 (0.9)	<.001		ns	7	1	48
Social conc.	ΔTreatment	PD crit	-0.63 (0.05)	<.001	0.01 (0.003)	0.02	17	2	135
Social conc.	ΔTreatment	AVPD		ns		ns	16	4	121
		BPD	-10.5 (0.9)	<.001	0.14 (0.05)	0.002			
Social conc.	ΔTreatment	BPD crit	-1.64 (0.2)	<.001	0.03 (0.01)	<.001	24	8	198
		NPD crit	-3.77 (0.5)	<.001	0.07 (0.03)	0.02			
		AVPD crit		ns	-0.02 (0.01)	0.03			
Relationalc.	ΔTreatment			ns	-0,1 (0.04)	0.04	0	0	4
Relationalc.	ΔTreatment	PD crit	-0.16 (0.04)	<.001		ns	2	0	19
Relationalc.	ΔTreatment	AVPD	-5.20 (0.7)	<.001	-0.09 (0.04)	0.03	6	0	54
		BPD		ns		ns			
Relationalc.	ΔTreatment	BPD crit		ns		ns	9	2	60
		NPD crit		ns		ns			
		AVPD crit	-1.15 (0.2)	<.001		ns			
Responsibl.	ΔTreatment		-4.6 (0.9)	<.001		ns	4	0	24
Responsibl.	ΔTreatment	PD crit	-0.4 (0.05)	<.001		ns	8	3	55
Responsibl.	ΔTreatment	AVPD	-3.2 (1.0)	0.002		ns	7	5	51
		BPD	-5.9 (0.9)	<.001	0.09 (0.04)	0.01			
Responsibl.	ΔTreatment	BPD crit	-0.92 (0.2)	<.001	0.02 (0.01)	0.002	9	13	71
		NPD crit	-1.9 (0.6)	<.001		ns			
		AVPD crit		ns	-0.02 (0.01)	0.02			

Demonstrates LMM estimations with standard errors (SE) for baseline levels (intercept estimate), and monthly change-rate (slope estimate), and corresponding variance estimates for five dependent variables (SIPP- SF domains). Significant estimates of variation are indicated by * (p<0.01). Goodness of model fit is indicated by Akaike Information Criterion (AIC), where smaller is better. Predictor analyses include the difference (Δ estimate) between treatment (ΔTreatment = Non-Manualized Treatment versus Mentalization-based Treatment), total number of PD criteria (crit), and the specific personality disorders, avoidant (AvPD) and borderline (BPD) in separate models and models controlling for the two. Interaction between ΔTreatment and PDs (moderators) additionally includes a model with the number of criteria within specific PDs with significant interaction. Non-significant differences are indicated by ns (*p*>0.05).

### Longitudinal change

#### Personality functioning

**Figure 1:**
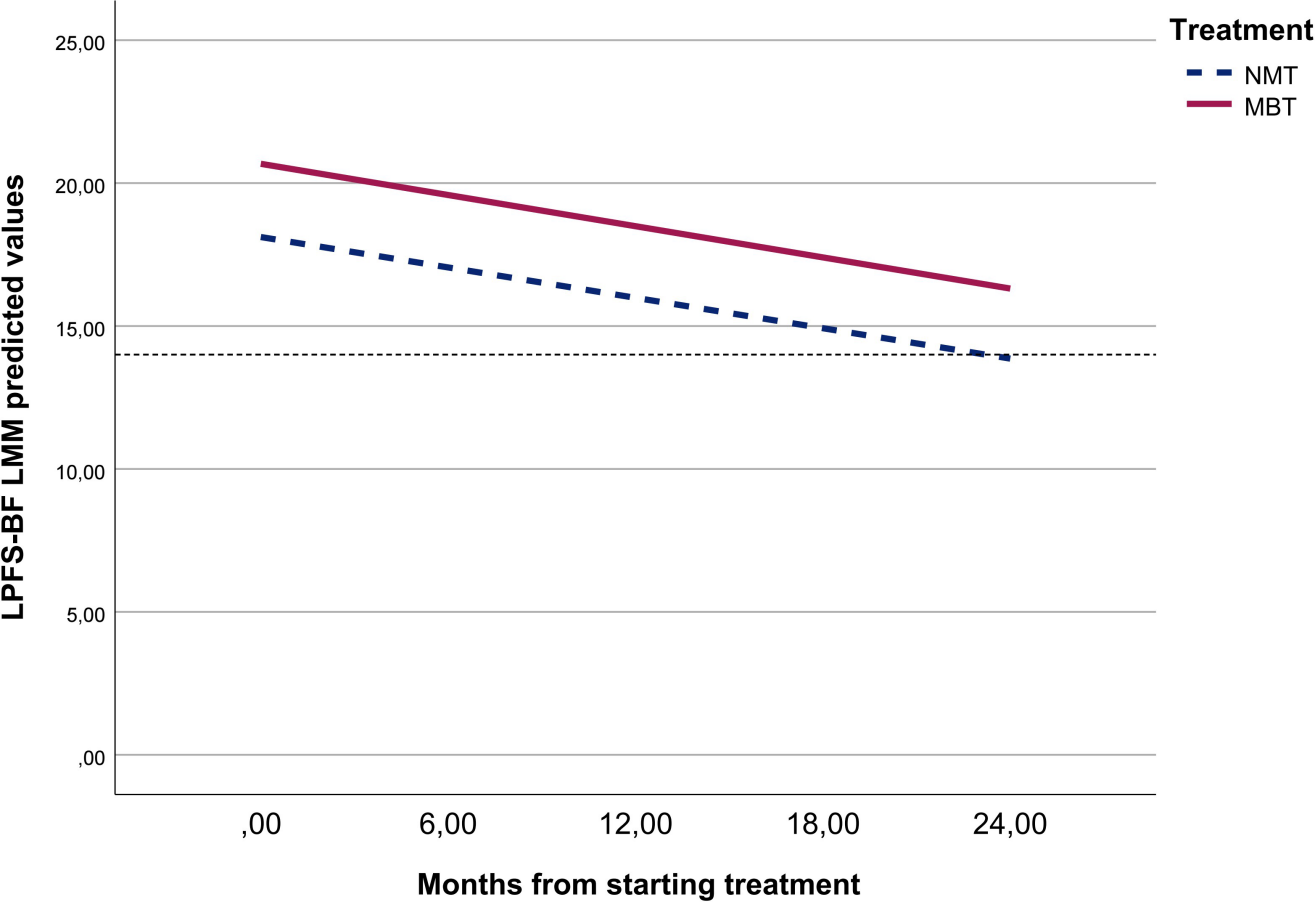
Initial levels and change of LPFS-BF relative to treatment. illustrates longitudinal trajectories based on LMM predicted values in accordance with estimates in **Table 3**. Non-Manualized Treatment (NMT) is compared to Mentalization-based Treatment (MBT). The difference in level of problems at the start of treatment was significant (*p*_LMM_< 0.05).The horizontal dotted reference line refers to LPFS-BF score of 14. Scores over 14 indicate clinically significant problems of personality functioning.

**Figure 2:**
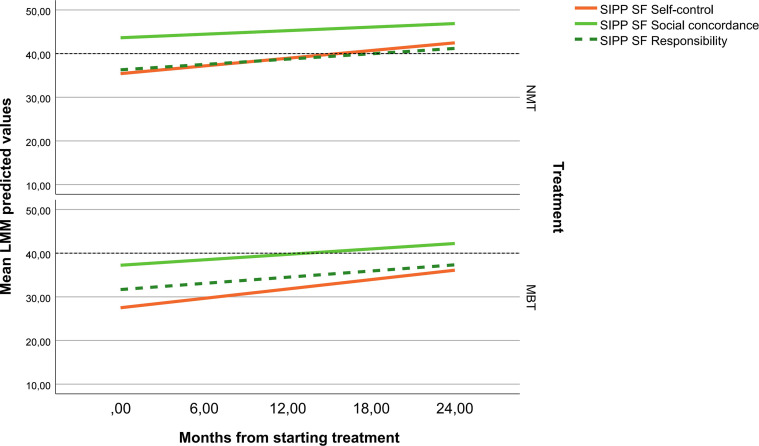
Trajectories of self-control, social concordance and responsibility. illustrates longitudinal trajectories (LMM predicted values) for three of the SIPP-SF domains in accordance with **Table 4** estimates. Non-Manualized Treatment (NMT) is compared to Mentalization-based Treatment (MBT). Differences in level of problems at the start of treatment were significant (*p*_LMM_< 0.05). The horizontal dotted reference line refers to a T-score of 40. Scores below 40 indicate clinically relevant personality problems.

**Figure 3:**
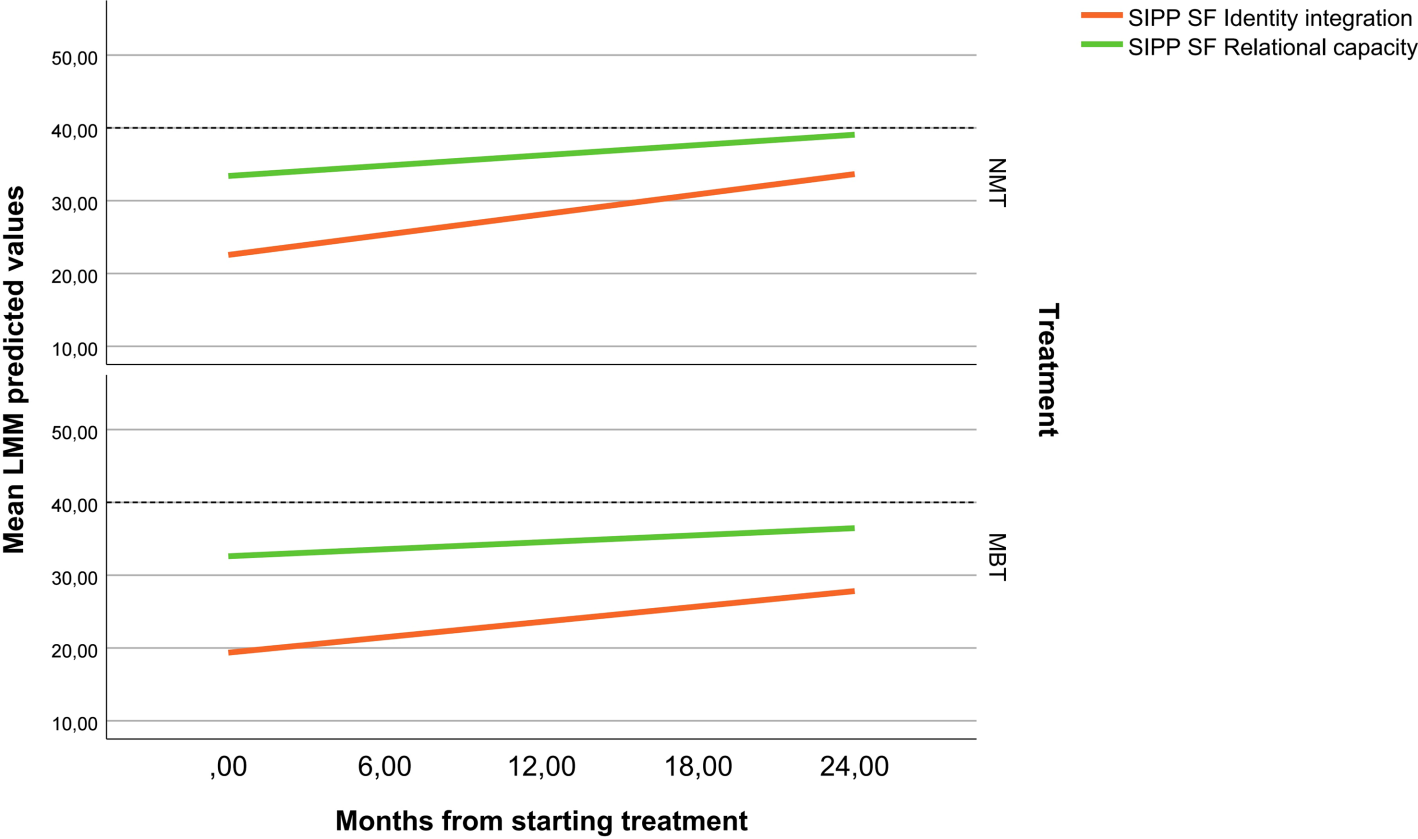
Trajectories of identity integration and relational capacity. illustrates longitudinal trajectories (LMM predicted values) for two of the SIPP-SF domains in accordance with **Table 4** estimates. Non-Manualized Treatment (NMT) is compared to Mentalization-based Treatment (MBT). Differences in level of problems at the start of treatment were significant as were differences in change over time (*p*_LMM_< 0.05). The horizontal dotted reference line refers to a T-score of 40. Scores below 40 indicate clinically relevant personality problems.

Longitudinal improvement during treatment was significant for all PF variables across both groups. Starting at different levels of functioning, MBT-NMT differences in longitudinal rates of change for LPFS-BF and the SIPP-SF domains Self-control, Social concordance and Responsibility were not significant (**Tables 3**, **4**; **Figures 1**, **2**). Longitudinal LMM effect sizes were large for LPFS-BF (MBT *d* = 1.0, NMT *d* = 0.9) and medium to large for *Self-control* (MBT *d* = 0.8, NMT *d* = 0.6), in the small range for *Social concordance* (MBT *d* = 0.4, NMT *d* = 0.3), and medium range for *Responsibility* (MBT *d* = 0.5, NMT *d* = 0.5). The proportion patients with LPFS-BF>14 was reduced by 27% in MBT and 41% in MNT (24-month LMM predicted values). For the SIPP-SF domains Identity integration and Relational capacity change-rates were poorer in MBT (**Table 4**; **Figure 3**). Longitudinal LMM effect-sizes were generally large for Identity integration (MBT *d* = 1.1, NMT *d* = 1.5), but small and medium for Relational capacity (MBT *d* = 0.4, NMT *d* = 0.7). In analyses controlling for differences in treatment duration, MBT-NMT differences were no longer significant for the SIPP SF domains Identity integration and Relational capacity (p>0.05). In LMM analyses investigating MBT-NMT differences, treatment conditions explained minimal variance in change over time (**Tables 3**, **4**). The total models explained 11% LPFS-BF variation, 12% SIPP-SF Self-control, 12% Identity integration, 6% Social concordance, 5% SIPP-SF Responsibility, and 4% Relational capacity (marginal pseudo R^2^).

#### Improvement of self-harm and suicide attempts

Among patients who reported self-harm last six months before treatment, self-harm remitted in approximately half the sample (MBT: 49%, NMT: 56%, *p*_NMT-MBT_ >0.05). Among patients who reported suicide attempts the last six months before treatment, the majority in MBT recorded no suicide attempts in the last 6 months of treatment (MBT: 83%), in NMT no patients reported suicide attempts in the last six-month phase of treatment (NMT:100%, *p*_NMT-MBT_ > 0.05).

#### Symptoms of depression and anxiety

Longitudinal improvement of symptom distress during treatment was significant (PHQ-9: slope estimate: -0.2, SE 0.01, p<0.001, GAD-7 slope estimate: -0.1, SE 0.01, *p*_LMM <_0.001). Symptoms improvement did not differ significantly by MBT-NMT for GAD-7 (*p*>0.05) and PHQ-9 (*p*>0.05). Effect-sizes were moderate to large for GAD-7: MBT *d* = 0.7, NMT *d* = 0.8, and large for PHQ-9: MBT *d* = 1.3, NMT *d* = 1.7).

#### Differences associated with PD status

A higher number of PD criteria, the presence of AvPD or BPD were associated with poorer baseline levels of LPFS-BF sum-score and independent of treatment, BPD was significantly associated with greater improvement (*p*_LMM_ < 0.01). Comparing improvements in MBT - NMT, improvement of LPFS-BF was significantly greater in MBT for patients with BPD. (**Table 3**). In exploratory analyses of PD criteria, the most elevated baseline LPFS-BF levels were associated with higher numbers of Avoidant, Narcissistic, and Borderline PD criteria (*p*_LMM_ <0.01). These were therefore included in separate models (**Tables 3**, **4**). The investigation of narcissistic PD criteria revealed no significant associations with LPFS-BF improvement over time. A higher number of AvPD criteria were associated with significantly greater improvement rate in NMT (**Table 3**).

A higher number of PD criteria was associated with poorer levels of baseline functioning across all models (*p*_LMM <_0.01). The presence of AvPD or BPD was associated with poorer baseline levels of SIPP SF Identity integration and Responsibility, BPD was also associated with poorer baseline levels of SIPP-SF *Self-control* and *Social concordance* and AVPD with poorer *Relational capacities* (*p*_LMM_ <0.01). Comparing improvements in MBT - NMT, improvement of SIPP-SF Self-control, Social concordance and Responsibility was significantly greater in MBT for patients with BPD (**Table 4**). Poorer improvement of SIPP-SF Self-control, Identity integration and Relational capacity was associated with AVPD in MBT (**Table 4**). The investigation of NPD criteria revealed only one significant association indicating greater improvement of Social concordance in MBT as compared to NMT (**Table 4**).

Models investigating MBT - NMT differences and PD variables explained minimal further variation (marginal pseudo R^2^). Models including the interaction between treatment and total number of Avoidant, Narcissistic, and Borderline PD criteria explained the most slope variation for SIPP SF subscales (mean 8%, highest Social concordance 13% and Self-control 12%, lowest Relational capacities 2%).

## Discussion

The present study investigates how treatment is applied in a regular clinical mental health outpatient service, specializing in treatment for patients with PD. The main focus was on personality functioning (PF). Comparing two structurally contrasting treatment conditions, MBT and NMT, the results highlight characteristics of patients at intake, development of PF during treatment, and how initial PD status relates to PF and outcome over time. Taken together, the results highlight PF as a credible marker for PD treatment selection but also signals shortcomings of only using global severity assessment when allocating patients to treatment. The main results of the study are discussed in the following.

### MBT recruited patients with the greater overall impairment of PF

Not surprisingly and in line with the originally specified target group, MBT patients were more often diagnosed with BPD and had a larger number of BPD criteria. Likewise, although the level of self-injury and suicidal behavior was noteworthy in both treatments, it was higher in MBT. Moreover, patient self-reports on PF generally indicated the largest differences in levels of Self-control. The results thus support a tendency of allocating patients to treatments allegedly tailored to their specific PD problems with referral to the documented effects (17). Overall severity of conditions was also reflected in the reports of former health service contact. MBT recruited slightly younger patients with earlier age of first contact with health services. A larger proportion reported experiences of hospital admissions. Previous research also shows that severe personality pathology often has onset in early years (74).

Focusing on overall severity across disorders, the tendency of greater complexity of disorders; comorbidity and symptom distress, among MBT patients found in the present study, expands the picture of MBT applied only for patients with BPD. We found that approximately one third qualified for AvPD, though proportions were somewhat higher in NMT. These findings were consistent across interview based diagnostic assessments performed by trained clinicians and patient self-reports on all aspects of PF, symptom distress and self-harming behaviors.

Former MBT studies have indicated that such specialized treatment programs are suited for the more complex BPD patients with additional personality pathology and symptomatic distress (31, 32). In a study comparing MBT in a day hospital or outpatient format, the patients with extensive traumatic backgrounds benefitted more in the intense format (75). Furthermore, in a naturalistic Danish study, differences in patients’ characteristics and allocation to “basic” or specialized treatment was explored (76). Corresponding to the present study, patients with more severe pathology, lower psychosocial functioning, and younger age were selected for the specialized treatment.

A main perspective addressing the first aim of the present study is how regular health services with MBT programs meet a broad range of PD patients and tend to allocate both the target group of BPD as well as patients with more severe impairment of PF across specific disorders to these comprehensive treatment programs.

### Minimal differences in PF improvement

The study compares different treatment approaches and includes a pragmatic level of quality assessment concerning the applied manualized treatment, MBT. It is often argued that implementation of manualized programs may be demanding (77). However, the present study signals a relatively high quality of the organization of MBT, well-trained therapists and acceptable adherence to MBT according to the applied manual in Norwegian language (21). The MBT teams had generally implemented regular MBT supervision for therapists, though less extensive than manual recommendations. MBT units had additional availability for therapists to attend half yearly video-based, supervisory MBT seminars enabling discussion, calibration, and updating of competence across units.

Irrespective of treatment conditions, units in the Network followed a common set of personality-focused principles and had a common availability of conferences and workshops for clinicians in all units (42). The personality-focused principles included systematic assessment before starting treatment and regular progress feedback based on patient self-reports throughout treatment, thus facilitating a shared understanding of personality problems and agreement on therapy focus, both between therapists and patients and within the treatment teams. Although NMT did not represent a given manualized program, it would be systematically informed by such regular personality focused evaluations, and this represents some degree of structural focus. Moreover, although not applying specific PD manualized treatment, it is likely that therapist interventions would be generally influenced by current knowledge on PD and treatment principles. Therapist interventions may have been somewhat inspired by MBT or other manualized PD treatments, not least as most of the units offered both MBT and NMT and therapists conducted both approaches. Some spill-over effect is therefore likely, but further exploration of this possibility is outside the scope of the present study. Generally, as a PD informed treatment, NMT may have had similarities to the quite recently coined “guideline informed treatment”, focusing on the explicit, mutual cooperative stance in PD treatment (78). Both MBT and the approaches in NMT represent therapies specifying a certain line of thinking, a style of interventions, a framework and format of therapy. Clear therapeutic strategies may enhance positive outcomes (79). It is a possibility that systematic quality monitoring may in itself, enhance such strategic competence in therapists, and as such, represent a positive outcome effect irrespective of treatment approach.

In the present study, the main trend across personality measures was significant improvement of PF with effect sizes in the medium to large range. Starting at different levels, the two treatments had parallel patterns of change over time and differences were below levels of significance. This was demonstrated for the overall, global levels of PF, the specific aspects of Self-control, Social concordance, and Responsibility. Our results also point to a parallel improvement of symptoms of anxiety and depression. Former studies of the longitudinal course of PD similarly demonstrate parallel remission of personality disorder and co-occurring symptom disorders (80). Our findings thus indicate considerable potential for improvement despite different initial levels of functioning and especially underline the treatment potential for patients with more severe problems of personality functioning. However, they may seem to contrast several former studies linking aspects of PF to the therapeutic process and following outcome. Generally, the main message has been that poor PF can complicate treatment and conversely, better PF can render the patient more receptive to therapeutic work (15, 81, 82). In the present study, the parallel positive developments in MBT and NMT may have been facilitated by tailored allocation to MBT or NMT in accordance with severity of condition. Considering the need for health services to accommodate patients with disorders of varying severity and type, our results can thus be seen to support an organization enabling differentiation between specialized treatments and simpler, more flexibly organized group-based treatments.

Comparing the five aspects of SIPP-SF assessed in this sample, Identity integration was the most severely impaired aspect, its improvement was highly significant across conditions and rendered high effect sizes in MBT, though even higher in NMT. For the PF aspect of Relational capacity, initial level of impairment was clinically relevant. Nonetheless, longitudinal effect sizes were in the moderate-poor range in both conditions, though significantly poorer in MBT. It seems that these PF aspects were hard to address in both treatments, but particularly in MBT. Impairment of Relational capacities may often imply a more distanced pattern of attachment anxiety and avoidance (83). This SIPP-SF dimension does probably not cover the more overinvolved (or disorganized) attachment style typical among BPD patients, which are the original target group of MBT (84). It is possible that NMT may represent a higher degree of flexibility enabling greater adaptation of the therapy to the more distancing attachment strategies. In our study NMT also included therapy approaches addressing embodied emotions, physical and social activities and non-verbal expressions such as art therapy. This may have increased its potential to work with socially inhibited patients. A recent survey of therapists experiences in the Network, revealed greatest difficulty in handling patients with emotional avoidance, both in individual and group therapy (64). Therapists in the survey also reported far more countertransference reactions towards patients with other personality problems than those of BPD, indicating less therapeutic confidence and coping when confronting personality problems other than those matched with the original MBT approach (Ibid.).

It is also worth noticing that MBT included somewhat longer treatment duration (24 months) than NMT (21 months), and higher intensity with combination therapy as standard. These format differences could probably compensate for the complicating aspects of more severe PD pathology in MBT. Interestingly, in analyses controlling for differences in treatment duration, differences in change-rates for Identity integration and Relational capacity were no longer significant. Patients who received longer-term therapies were in both conditions associated with poorer functioning and also poorer rates of change.

Corresponding to the trends of improved PF, the study demonstrates remission of self-harming behaviors among approximately half of the patients who initially reported such problems. Self-harming behaviors and suicide attempts were considerably more frequent on admission in MBT. Nonetheless, corresponding proportions with remission were evident in MBT and NMT. However, a recent MBT study including a large sample (n=186) reported higher levels of current self-harm on admission and a 70% remission rate during treatment (66). Development of treatment refractory self-harm can be a severe health issue, its consequences should not be underestimated (85). Reasons for the lower self-harm remission rate in our study are uncertain. The referred MBT study was based on a tertiary level treatment unit, enabling a more homogenous patient sample of young adults with severe BPD, possibly suggesting positive group effects for teams, therapists, and patients when assembling patients with aligned problems. The results indicate limitations of both MBT and NMT implemented broadly within mental health services, suggesting a need for closer follow-up, possible on a tertiary level, and also better collaboration across service levels for the most severe self-harming patients (86). In contrast, a former report on psychodynamic group therapy treatment indicated only 35% remission of self-harm (87), thus suggesting that for the current NMT, where psychodynamic group therapy was frequent, self-harm management can be said to have improved. Former studies of patients in MBT have demonstrated the cost burden involved among patients with moderate to severe personality disorder and the health service cost savings associated with self-harm remission during treatment – emphasizing the reduced need for emergency services and acute hospitalization during and after specialized treatment (4, 88).

Our study thus suggests that in addition to evaluation of the level of PF, successful treatment allocation also includes individual consideration of how a manualized program is matched to the particular social vulnerabilities and emphasizes the need for maintaining a variety of treatment strategies and competence within mental health services.

### Differences according to PD status

The total number of PD criteria was mainly associated with the initial treatment allocation, and given such systematic distribution, variation in this aspect of PD severity was not a predictor of the improvement of global PF nor specific self-domains in MBT or NMT. For Social concordance, increasing numbers of PD criteria was, however, moderately associated with greater improvement over time in MBT. These results are in line with former research comparing psychodynamic group-based treatment programs and MBT which indicated that patients with more severe PD conditions had a greater benefit of MBT, whereas patients with BPD and little other comorbidity responded well in both treatments (31). This former group-based treatment program was conducted before the establishment of manualized PD treatments in Norway. It had a step-down design with an initial, intensive 18-week day hospital program followed by weekly long-term group therapy as an outpatient, stand-alone treatment. The present NMT is a flexible outpatient format incorporating stand-alone group treatment or combinations of groups or individual therapy. It should be noted that in our study, differences were minor. Based on the current results NMT seems to accommodate varying PD conditions better than the former group-based step-down program.

The presence of BPD or borderline PD criteria consistently favored MBT in terms of greater improvement of global PF and more specifically, Self-control, Social concordance and Responsibility. The domain of *Self-control* includes the capacity to tolerate, use and control emotions and impulses, aspects of PF matching patients with BPD and the original target of MBT. The active and engaged therapeutic attitude, where MBT therapists aim to regulate escalating and destructive processes through the sessions, includes a combination of curious and validating interventions and further exploration of mental states, which may contribute to increase mentalizing capacities associated with Self-control (89). The domain of Social concordance includes the ability to value someone’s identity and withhold aggressive impulses and cooperate with others. Taken together with the domain of Responsibility, the MBT group format will in particular challenge and explore such interpersonal functioning. The mentalizing therapeutic attitude represents and maintains authority, safeguards group boundaries, shared values and respect for each individual and their role in the group, while sustaining a not-knowing stance (22). The group requires regular attendance and adherence to rules through a cooperative process. The treatment provides ample exploration of situations, contexts, feelings, and intentions, and highlights differences between patients. Group participation may cultivate and expand capacities for interpersonal commitment, respect, attachment, explicit sharing, and challenge distrustful, negative interpretations.

The independent presence of AvPD or avoidant PD criteria favored NMT on all investigated PF aspects. Problems of Identity integration were common aspects of PD in general, highly significant for both AvPD and BPD. This domain involves coherence of identity, stable sense of self, feelings of meaning and joy. Qualitative research specifically exploring subjective experiences of having AvPD, mirror this finding. Patients with AvPD typically report a vague sense of self, and insecurity in own decisions and capacities (90). Our results indicate that AvPD patients had poorer improvements on such aspects in the MBT program. Identity problems in terms of alexithymia has in former studies been proposed as a marker of PF in AvPD (91). Poor capacities for identifying feelings have been reported across different PDs (44). Emphasizing relational vulnerability, in the sited study, AvPD was also associated with difficulties expressing feelings. Although several overlapping issues of identity, AvPD is in many ways different from BPD. The poorer course associated with AvPD in MBT may therefore be related to a suboptimal treatment framework and focus.

The association between impaired Relational capacity and AvPD was a significant finding in our study. Relational problems of AvPD include fear of becoming close to others, feelings of insecurity, anxiety and shame that hamper intimacy and experiences of authentic contact and meaningful relationships (90). The relational problems may have hampered their participation in MBT, not least when attending groups together with more extrovert (BPD) fellow patients. Furthermore, as our study demonstrates, problems of Self-control and Social concordance were less characteristic features of AvPD. With typical problems of negative self-esteem, perfectionism, self-inhibition and withdrawal, its clinical presentation is more likely to represent too much self-control (92).

AvPD criteria was associated with poorer development in the domain Responsibility. This may be considered at odds with the clinical impression of AvPD patients with high guilt and shame proneness, fear of doing wrong and sometimes perfectionistic tendencies. On the other hand, characteristic avoidance strategies may be reflected in poorer maintenance of goal-directed behaviors with avoidance of persisting obligations when fear and difficult emotions are triggered. The finding was limited to MBT, not apparent in NMT. Speculatively, it might reflect challenges in involvement or coming forth, during participation in the possibly more intense emotional and conflictual climate with BPD patients in MBT groups.

With respect to the high frequency of psychodynamic group therapies in NMT, our findings of favorable outcomes in NMT for relational capacities and AvPD may be somewhat surprising as traditional psychodynamic group treatment largely relies on patients’ own initiative to confront inhibitions, express themselves and share. It should also be noted that patients with AvPD in MBT had more severe conditions, often in combination with other PD criteria, which may exert an additional complicating factor in treatment (91) and accentuate suboptimal effects these patients had in MBT.

Narcissism is considered a multifaceted construct including sub-types of overt/extrovert (grandiose) and covert/introvert (vulnerable) expressions (93). The SCID-5- PD taps more of the overt type which was more frequent in the MBT condition. Our study demonstrates that a higher number of narcissistic criteria were mainly associated with more problems of Responsibility and Social concordance. Irresponsible or unsocial behaviors are known to represent problems in therapy, perhaps more so in an NMT format. In our study, the presence of narcissistic criteria favored MBT in terms of significantly greater improvement in Social concordance. One could speculate that the manualized format of MBT-groups helps distribute time of attention for each patient and makes themes of social dominance, competition, envy, and devaluation more manageable. In addition, the relational difficulties NPD patients experience in adapting and attaching to groups may be helped by the individual therapy where they have opportunities to process and be motivated to continue group engagement. In a recent study, including a mixed sample of outpatients (in forensic centers) and community, patients with narcissism showed that increase in both Identity integration and Social concordance where positive moderators of *Relational capacities* (94). In light of recent discussions on possible under recognition of narcissistic traits, the present findings add to understanding of their clinical significance.

The improvements among patients with PDs demonstrated in both MBT and the more varied NMT is good news in this broad context of PD mental health services across urban and rural settings. These findings suggest that the full MBT program can be successfully implemented, thus providing highly specialized treatment for patients with substantial personality problems, BPD and complicating features such as narcissistic traits. Furthermore, the application of NMT is a good alternative among patients with more moderate conditions. In addition, it accommodates conditions requiring a more tailored approach. Our study underpins different needs concerning treatment approach, especially for BPD and AvPD, and indicates that selection of treatment based on overall severity of PD alone is not sufficient.

Tailoring of treatment for AvPD is currently a relevant research focus, several studies have piloted promising interventions (95–99) but only a few treatments have been tested in RCTs (100, 101). Despite a lack of evidence-based interventions, increasing realization of AvPD problems in available PD treatments and interest in development of specialized AvPD treatment is evident among clinicians (102). Our results confirm the need for specific attention to treatment for patients with such personality problems.

## Strengths and limitations

The naturalistic design is in accordance with the overall study aim seeking to explore utility of treatment applied within health services for patients with PD. It is therefore a particular strength of this study that data reflects treatments delivered in regular specialist mental health services across different geographical regions. The sample reflects the distribution of personality problems among patients seeking treatment within PD mental health services. However, it does not represent all types of PDs and does not include all mental health services in Norway. Furthermore, patients were primarily young adults and mostly female. In further accordance with the stated objectives, the data sample is large and includes detailed clinical information relevant for the study of PD. However, this design does not allow for causal interpretations. Unmeasured factors such as clinician preference and organizational differences between sites may also have influenced treatment assignments and limits causal interpretations of comparative outcome trajectories. The variety of treatments in NMT may represent a strength for local tailoring but represents a methodological limitation concerning unmeasured variation in this naturalistic design. Further investigation of longitudinal variation associated with the different modalities in NMT is outside the scope of the present study. It should also be noted that the present study investigates MBT and did not investigate other types of manualized PD treatments known to be effective, especially for patients with BPD.

Due to the design of the study, it lacks tests of diagnostic reliability. However, the regular diagnostic procedures hold a high standard as they are conducted by qualified health professionals and supported by use of systematic, well validated instruments and availability of training for clinicians. Assessment of experiential, personal qualities include both interviews and patient self-reports (103). The data is thus more personality focused and assessments hold a higher standard and level of detail than more general data retrieved from Norwegian Patient Registries.

Data is based on local administration of measures and incomplete datasets are to be expected in clinical studies with this design. The longitudinal organization of data is nonetheless a strength and to ensure maximum utilization of all available data, the study is based on LMMs statistics. The method enables utilization of unbalanced longitudinal data over long study periods (73).

A strength in our study is the evaluation of MBT quality, both regarding therapy and organizational structure. The organizational quality aspect is rarely monitored in studies of MBT, despite high clinical relevance. Possible limitations of the general validity of quality ratings are that in-session MBT adherence ratings were performed as a regular, supervisory clinical practice and may have had some overrepresentation of therapies the clinicians found problematic, and that organizational quality was assessed in retrospect. Across MBT units, the organizational quality of MBT was generally high.

Data anonymity research regulations of the quality register did not allow direct coupling of site-specific or therapist specific quality information to specific patients and the data set as a whole. The study enquired about standard MBT and included no further specification of other MBT adaptations. Study permissions in this project did not allow for assessment of variation of MBT in-session quality across specific MBT units. On a therapist level, mean in-session MBT adherence ratings with their standard deviation indicated some therapist variation, but levels were still within an acceptable score-range.

As NMT represented a heterogenous, non-manualized treatment condition, there were no adherence measures and its format and approach was thus more heterogenic across treatment sites than MBT. Though the group format and psychodynamic group therapy was a dominating aspect, NMT should be considered a treatment tailored in accordance with local resources and patient factors, and not as a specific treatment.

## Conclusions

Patients selected for MBT had significantly more severe impairment of PF and more often complex conditions including self-harm, suicide attempts, comorbid mental disorders, and higher burden of symptom distress. Starting at significantly different levels of PF, improvement during treatment represented medium to large effect sizes, and differences according to treatment condition were minor. Total number of PD criteria as a marker of PD severity was significantly associated with allocation to MBT or NMT, but controlling for this baseline variation, did not predict the longitudinal course of most PF aspects. Borderline criteria and narcissistic criteria were associated with greater improvement in MBT. Conversely, avoidant criteria were associated with poorer improvement in MBT. The study identified Relational capacity as a central aspect of PF particularly associated with AvPD and with poorer improvement in MBT. Our findings suggest that PF can function as a coarse-grained marker when matching patients and treatment, and it supports the need for differentiated treatment offers for patients with different levels of PF in mental health services. The study also underpins the need for further development of tailored treatment addressing frequently occurring relational personality problems often associated with AvPD.

## Data Availability

The datasets presented in this article are not readily available because it originates from a quality register of the Norwegian Network for Personality Disorders. Owing to restrictions related to patient confidentiality, data can only be accessed upon specific request. Requests to access the datasets should be directed to e.h.kvarstein@medisin.uio.no.
