# Supplementary material for: Improvement of personality functioning in patients with personality disorders: a comparative study of mentalization-based treatment versus non-manualized treatments
Source: Front Psychiatry. 2026 Jun 22;17:1831782. doi: 10.3389/fpsyt.2026.1831782 (PMC13333664; doi:10.3389/fpsyt.2026.1831782)
Supplement: Supplementary file 1 [file Table1.docx]

***Supplementary material***

**Improvement of personality functioning in patients with personality disorders: A comparative study of mentalization- based treatment versus non-manualized treatments**

Kjetil Bremer*, Geir Pedersen, Andreas Ekberg, Katharina T. E. Morken, Helene A. Nissen -Lie, Eileen Oftedal, Theresa Wilberg, Elfrida H. Kvarstein

***Correspondence:**

Kjetil.bremer@ous-hf.no

**Supplement 1: Patient-report of former treatment, self-harm and suicide attempts**

| **Former outpatient treatment** | **Answer option** |
| --- | --- |
| How old were you when fist contacting health care concerning mental health difficulties (school health service, doctor, psychologist, hospital and the like)? | Before 13 years old, 13-18 years old, 19-29 years old, I was in the thirties, I was in the forties, I was in the fifties or older. |
| Have you had earlier periods of treatment for mental health difficulties? | Yes/no. |
| *If yes*: How many treatment periods? | Just one treatment period, two treatment periods, three or more treatment periods. |
| **Former inpatient treatment** |  |
| Have you had former inpatient admissions? | Yes/no. |
| *If yes:* How many inpatient admissions in total? | Just once, twice, more than two times. |
| **Self-harm** |  |
| Have you ever on purpose harmed yourself? (eg cutting, burning, headbanging etc) | Yes/no. |
| Have you within the last 6 months on purpose harmed yourself? | Yes/no. |
| **Suicide attempts** |  |
| Have you ever purposely tried to kill yourself? | Yes/no. |
| Have you within the last 6 months purposely tried to kill yourself? | Yes/no. |

**Supplement 2: Statistics and analysis**

The main statistical method was Linear mixed models (LMM) ([Heck et al. (2013)](#_ENREF_18) (Singer & Willett, 2003). Variables of PF (LPFS-BF sum-score and the five SIPP- SF domains) were analyzed as dependent variables. Treatment (MBT or NMT) was treated as an independent variable (predictor). Time (months from baseline) was modelled as a continuous variable. In accordance with log likelihood estimations, the best-fitted model included linear time, random intercept and slope, and unstructured covariance. Pseudo-R^2^ measures ranged 1-6% (marginal R^2^) in the six models when specifying only fixed effects and improved in all models with random coefficients: LPFS-BF model, marginal: 8%, conditional: 74%, SIPP-SF models, *Self-control* marginal: 6%, conditional: 82%, *Identity Integration* marginal: 10%, conditional: 77%, *Relational Capacity* marginal: 4%, conditional: 78%, *Social Concordance* marginal: 2%, conditional: 80%, *Responsibility* marginal: 3%, conditional: 77%. We report LMM estimates for change trajectories (intercept and slope), predictor-associated deviation, variance components (intercept and slope), overall pseudo R^2^ measures and log likelihood statistics (Akaike’s information Criterion, AIC). Explained variance is the % reduction of variance estimates from the model without predictor (reference value). Strong inferences are indicated by p < 0.01 (fixed effects), % explained variance, and improved model fit (Akaike’s Information Criterion, AIC). Exact p-values are reported when p<0.05. Additional corresponding models included the dependent variables PHQ-9 and GAD7.

The models are generally specified in the formula:

Variables of PF = Fixed Intercept + (Fixed Effect: Treatment) + (Fixed Effect: Time) + (Random Intercept) + (Random Slope * Time) + Error.

Differences according to PD status were further investigated in separate PF models adding as fixed effects: PD variable * Treatment + PD variable * Treatment * time. Due to considerable comorbidity of PD features, these models included 1) the two most frequent PD categories (Borderline and avoidant PD), 2) the total number of PD criteria and 3) independent contributions of specific PD criteria

**Supplement 3: Control analysis**

A large proportion had comorbid mood disorders and corresponding self-reported symptoms (PHQ-9) were high. Investigation of models (LPFS-BF and all SIPP-SF domains) with treatment condition as predictor, controlling for possible effects of comorbid mood disorder, did not change main results. Mood disorder was associated with poorer baseline SIPP-SF self-control and Identity Integration levels (p<0.05), but was not associated with deviating change in any models (p>0.05).

The majority of patients were female, in a young adult age group (age < 31 years: 68%). Younger age and male gender were associated with poorer personality functioning at baseline. Deviating longitudinal change was non-significant except for a significant gender effect for SIPP SF self-control with slower change associated with female gender, p=0.01). LMM main analyses with treatment condition as predictor, controlling for gender and age, did not change main results.

Patients who had received MBT and additional NMT in the study period were associated with significantly poorer baseline levels. When controlling for possible effects of additional therapy, LMM main trends of change remained unchanged.

LMM analyses were replicated in samples including only patients with PD. Main trends remained.

Differences in treatment duration was an additional source of differences in the number of assessments. Mean duration in MBT was 24 months (SD 12). Mean duration in NMT was 21 months (SD 12). In analyses controlling for differences in treatment duration, MBT-NMT differences were no longer significant for the SIPP SF domains, *Identity integration* and *Relational Capacity* (p>0.05). Patients who received longer-term therapies were in both conditions associated with poorer functioning and also poorer rates of change. However, effects of treatment duration differences were generally small and models adding treatment duration explained no further sample variation in all PF aspects (marginal pseudo R^2^).
